# Supplementary material for: Genome-Wide Identification of Barley ABC Genes and Their Expression in Response to Abiotic Stress Treatment
Source: Plants (Basel). 2020 Sep 28;9(10):1281. doi: 10.3390/plants9101281 (PMC7599588; doi:10.3390/plants9101281)
Supplement: Supplementary file 1 [file plants-09-01281-s001.pdf]

Figure S1

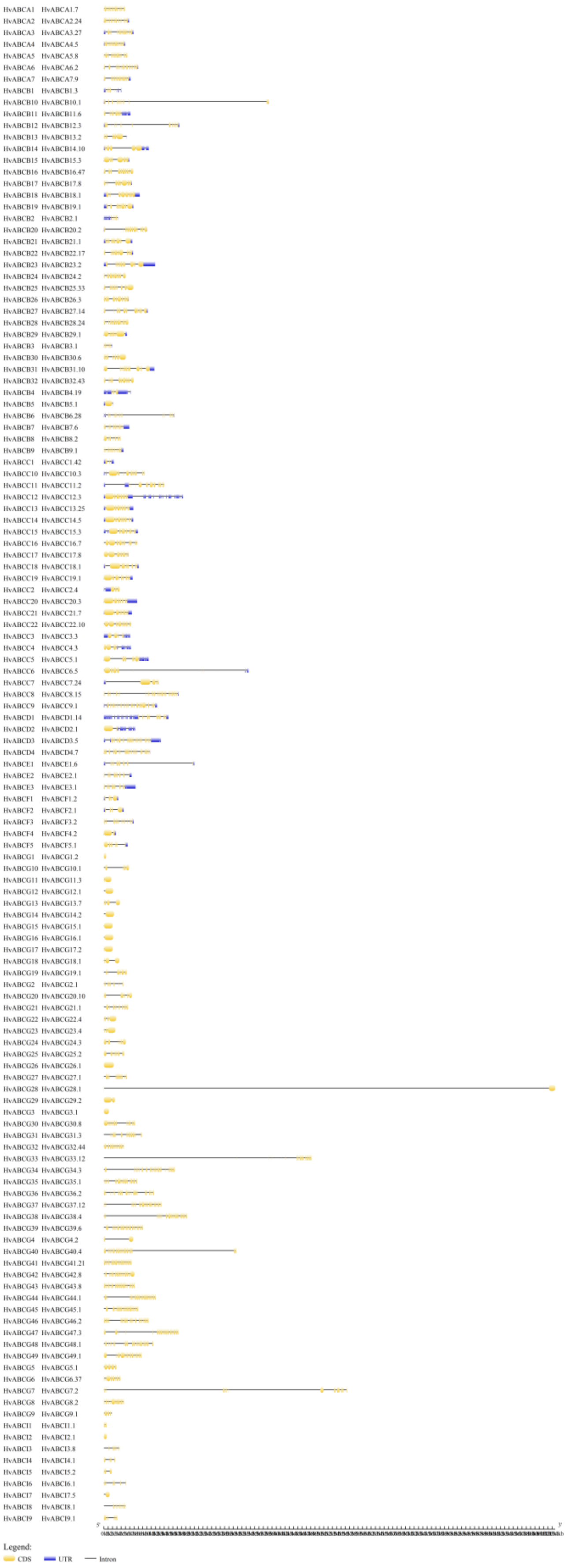
HvABCA1 HvABCA1.7
HvABCA2 HvABCA2.24
HvABCA3 HvABCA3.27
HvABCA4 HvABCA4.5
HvABCA5 HvABCA5.8
HvABCA6 HvABCA6.2
HvABCA7 HvABCA7.9
HvABCB1 HvABCB1.3
HvABCB10 HvABCB10.1
HvABCB11 HvABCB11.6
HvABCB12 HvABCB12.3
HvABCB13 HvABCB13.2
HvABCB14 HvABCB14.10
HvABCB15 HvABCB15.3
HvABCB16 HvABCB16.47
HvABCB17 HvABCB17.8
HvABCB18 HvABCB18.1
HvABCB19 HvABCB19.1
HvABCB2 HvABCB2.1
HvABCB20 HvABCB20.2
HvABCB21 HvABCB21.1
HvABCB22 HvABCB22.17
HvABCB23 HvABCB23.2
HvABCB24 HvABCB24.2
HvABCB25 HvABCB25.33
HvABCB26 HvABCB26.3
HvABCB27 HvABCB27.14
HvABCB28 HvABCB28.24
HvABCB29 HvABCB29.1
HvABCB3 HvABCB3.1
HvABCB30 HvABCB30.6
HvABCB31 HvABCB31.10
HvABCB32 HvABCB32.43
HvABCB4 HvABCB4.19
HvABCB5 HvABCB5.1
HvABCB6 HvABCB6.28
HvABCB7 HvABCB7.6
HvABCB8 HvABCB8.2
HvABCB9 HvABCB9.1
HvABCC1 HvABCC1.42
HvABCC10 HvABCC10.3
HvABCC11 HvABCC11.2
HvABCC12 HvABCC12.3
HvABCC13 HvABCC13.25
HvABCC14 HvABCC14.5
HvABCC15 HvABCC15.3
HvABCC16 HvABCC16.7
HvABCC17 HvABCC17.8
HvABCC18 HvABCC18.1
HvABCC19 HvABCC19.1
HvABCC2 HvABCC2.4
HvABCC20 HvABCC20.3
HvABCC21 HvABCC21.7
HvABCC22 HvABCC22.10
HvABCC3 HvABCC3.3
HvABCC4 HvABCC4.3
HvABCC5 HvABCC5.1
HvABCC6 HvABCC6.5
HvABCC7 HvABCC7.24
HvABCC8 HvABCC8.15
HvABCC9 HvABCC9.1
HvABCD1 HvABCD1.14
HvABCD2 HvABCD2.1
HvABCD3 HvABCD3.5
HvABCD4 HvABCD4.7
HvABCE1 HvABCE1.6
HvABCE2 HvABCE2.1
HvABCE3 HvABCE3.1
HvABCF1 HvABCF1.2
HvABCF2 HvABCF2.1
HvABCF3 HvABCF3.2
HvABCF4 HvABCF4.2
HvABCF5 HvABCF5.1
HvABCG1 HvABCG1.2
HvABCG10 HvABCG10.1
HvABCG11 HvABCG11.3
HvABCG12 HvABCG12.1
HvABCG13 HvABCG13.7
HvABCG14 HvABCG14.2
HvABCG15 HvABCG15.1
HvABCG16 HvABCG16.1
HvABCG17 HvABCG17.2
HvABCG18 HvABCG18.1
HvABCG19 HvABCG19.1
HvABCG2 HvABCG2.1
HvABCG20 HvABCG20.10
HvABCG21 HvABCG21.1
HvABCG22 HvABCG22.4
HvABCG23 HvABCG23.4
HvABCG24 HvABCG24.3
HvABCG25 HvABCG25.2
HvABCG26 HvABCG26.1
HvABCG27 HvABCG27.1
HvABCG28 HvABCG28.1
HvABCG29 HvABCG29.2
HvABCG3 HvABCG3.1
HvABCG30 HvABCG30.8
HvABCG31 HvABCG31.3
HvABCG32 HvABCG32.44
HvABCG33 HvABCG33.12
HvABCG34 HvABCG34.3
HvABCG35 HvABCG35.1
HvABCG36 HvABCG36.2
HvABCG37 HvABCG37.12
HvABCG38 HvABCG38.4
HvABCG39 HvABCG39.6
HvABCG4 HvABCG4.2
HvABCG40 HvABCG40.4
HvABCG41 HvABCG41.21
HvABCG42 HvABCG42.8
HvABCG43 HvABCG43.8
HvABCG44 HvABCG44.1
HvABCG45 HvABCG45.1
HvABCG46 HvABCG46.2
HvABCG47 HvABCG47.3
HvABCG48 HvABCG48.1
HvABCG49 HvABCG49.1
HvABCG5 HvABCG5.1
HvABCG6 HvABCG6.37
HvABCG7 HvABCG7.2
HvABCG8 HvABCG8.2
HvABCG9 HvABCG9.1
HvABCI1 HvABCI1.1
HvABCI2 HvABCI2.1
HvABCI3 HvABCI3.8
HvABCI4 HvABCI4.1
HvABCI5 HvABCI5.2
HvABCI6 HvABCI6.1
HvABCI7 HvABCI7.5
HvABCI8 HvABCI8.1
HvABCI9 HvABCI9.1
5'
3'
Legend:
CDS
UTR
Intron

Figure S2

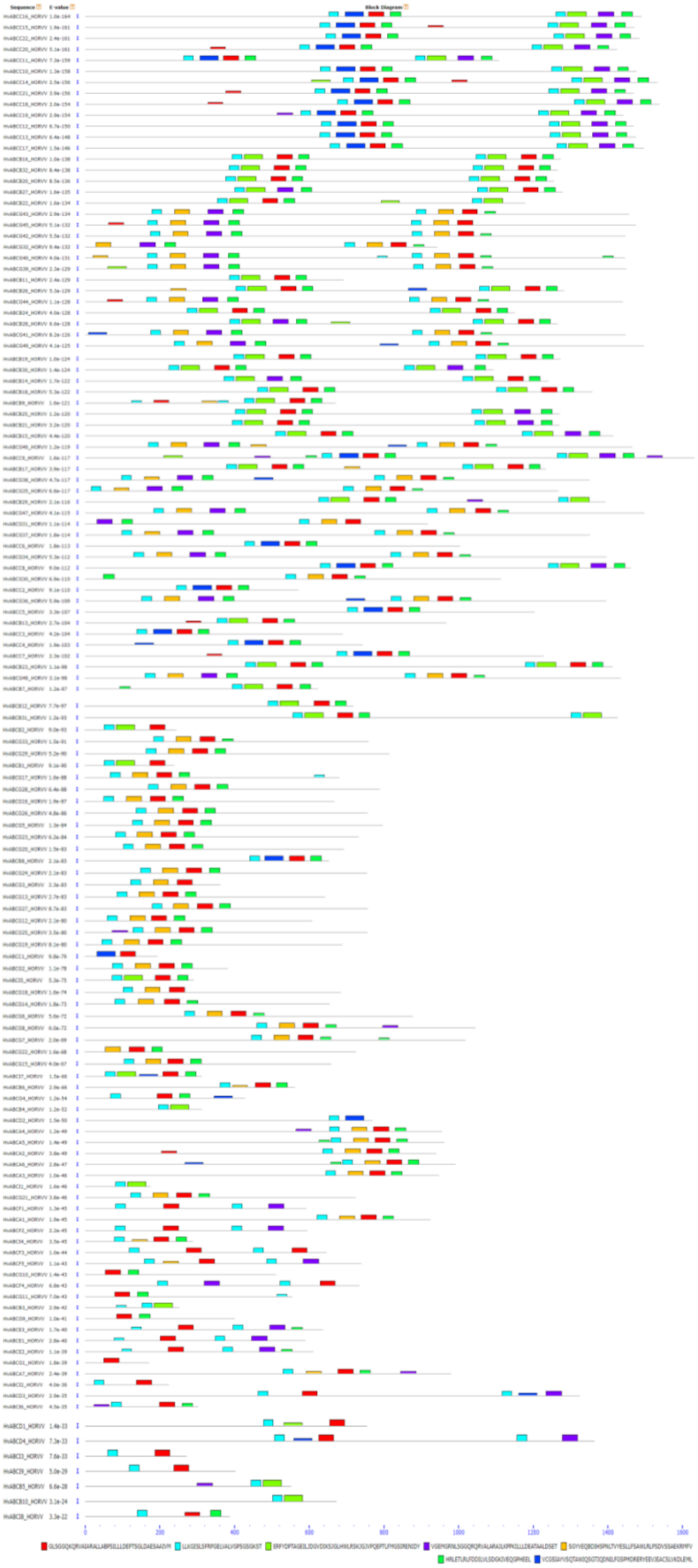

Table S1 Physicochemical properties、domain and subcellular localization of ABC proteins in barley

Gene name	Protein name	Type of domain	No.of exons	ORF(aa)	MW(kD)	PI	Subcellular localization
HvABCA1	A0A287G9Z1	NBD	18	923	102687.69	9.05	Plasma membrane
HvABCA2	A0A287TZ07	TMD2-NBD	17	939	104338.35	7.49	Plasma membrane
HvABCA3	M0WZM9	TMD2-NBD	18	946	105752.77	8.22	Plasma membrane
HvABCA4	A0A287G9Z0	TMD2-NBD	18	954	105599.25	8.75	Plasma membrane
HvABCA5	A0A287Q0T9	TMD2-NBD	18	960	106384.45	8.96	Plasma membrane
HvABCA6	A0A287X621	TMD2-NBD	18	991	110383.64	8.98	Plasma membrane
HvABCA7	A0A287GA22	TMD2-NBD	15	979	108695.01	7.84	Endoplasm. retic.
HvABCB1	A0A287WGF7	NBD	12	239	26033.42	5.53	Vacuolar
HvABCB2	A0A287HF47	NBD	14	245	27028.96	6.71	Vacuolar
HvABCB3	A0A287LPQ3	NBD	10	252	27438.15	9.67	Vacuolar
HvABCB4	A0A287LDX6	TMD-NBD	6	312	33689.02	6.76	Plasma membrane
HvABCB5	A0A287INY9	TMD-NBD	2	551	——	——	Plasma membrane
HvABCB6	A0A287GY78	TMD-NBD	19	561	61902.17	7.2	Mitochondrial

HvABCB7	A0A287MAM0	TMD-NBD	18	622	68825.58	8.3	Chloroplast
HvABCB8	A0A287IF94	TMD-NBD	10	653	70255.49	9.33	Chloroplast
HvABCB9	A0A287SDQ1	TMD-NBD	17	670	72073.96	9.06	Vacuolar
HvABCB10	A0A287P176	TMD-NBD	15	673	——	——	Mitochondrial
HvABCB11	A0A287LE15	TMD-NBD	13	692	74809.6	5.93	Plasma membrane
HvABCB12	A0A287XAI1	TMD-NBD	16	718	77500.28	8.92	Plasma membrane
HvABCB13	A0A287IP42	TMD-NBD	7	965	——	——	Plasma membrane
HvABCB14	M0VMJ6	TMD-NBD-TMD-NBD	7	1238	133704.44	7.57	Plasma membrane
HvABCB15	A0A287WPW5	TMD-NBD-TMD-NBD	6	1411	152919.97	8.71	Plasma membrane
HvABCB16	A0A287LE58	TMD-NBD-TMD-NBD	12	1273	137699.22	8.15	Plasma membrane
HvABCB17	A0A287LHW3	TMD-NBD-TMD-NBD	9	1231	133919.38	8.33	Plasma membrane
HvABCB18	A0A287UIA6	TMD-NBD-TMD-NBD	9	1355	148301.27	9.24	Plasma membrane
HvABCB19	M0X0N8	TMD-NBD-TMD-NBD	9	1270	137759.43	8.27	Plasma membrane
HvABCB20	A0A287GW34	TMD-NBD-TMD-NBD	12	1255	135477.62	5.96	Plasma membrane
HvABCB	A0A287ILD	TMD-NBD-TMD-NBD	10	1267	137459	8.6	Plasma membrane

21	6				.03	5	
HvABCB22	A0A287LEA0	TMD-NBD-TMD-TMD-NBD	11	1178	128217.97	9.02	Plasma membrane
HvABCB23	A0A287P8S5	TMD-NBD-TMD-NBD	11	1409	155449.34	6.02	Plasma membrane
HvABCB24	M0VMZ3	TMD-NBD-TMD-NBD	10	1151	124397.07	7.59	Plasma membrane
HvABCB25	A0A287WUM2	TMD-NBD-TMD-NBD	10	1268	139242.24	6.44	Plasma membrane
HvABCB26	A0A287ERQ6	TMD-NBD-TMD-NBD	10	1283	137505.48	7.05	Plasma membrane
HvABCB27	A0A287LDR1	TMD-NBD-TMD-NBD	12	1280	139374.41	7.36	Plasma membrane
HvABCB28	A0A287GDI0	TMD-NBD-TMD-NBD	12	1265	136185.54	6.09	Plasma membrane
HvABCB29	A0A287PQZ8	TMD-NBD-TMD-NBD	8	1391	148863.82	9.32	Plasma membrane
HvABCB30	A0A287E2C9	TMD-NBD-TMD-NBD	7	1091	118609.17	7.32	Plasma membrane
HvABCB31	A0A287MU70	TMD-NBD-TMD-NBD	11	1425	156509.67	8.08	Plasma membrane
HvABCB32	A0A287TK16	TMD-NBD-TMD-NBD	12	1265	136357.55	7.58	Plasma membrane
HvABCC1	A0A287XR02	NBD	3	195	21356.47	5.73	Plasma membrane
HvABCC2	A0A287VRA1	TMD-NBD	5	570	64348.36	7.95	Plasma membrane
HvABCC3	A0A287E8H3	NBD-TMD	10	689	76862.93	5.37	Plasma membrane

HvABCC4	A0A287G2E6	TMD-NBD	13	742	81878.76	6.45	Plasma membrane
HvABCC5	A0A287SQS9	TMD-NBD	11	1201	——	——	Plasma membrane
HvABCC6	A0A287VR07	TMD-NBD-TMD	13	1002	112321.95	9.24	Plasma membrane
HvABCC7	A0A287JB27	TMD-NBD-TMD	6	1226	137384.83	8.86	Plasma membrane
HvABCC8	A0A287VX18	TMD-NBD-TMD-NBD	34	1459	162273.12	7.75	Plasma membrane
HvABCC9	A0A287J912	TMD-NBD-TMD-NBD	29	1628	182783.15	7.47	Plasma membrane
HvABCC10	A0A287H0Y8	TMD-NBD-TMD-NBD	13	1477	——	——	Plasma membrane
HvABCC11	A0A287H0Z9	TMD-NBD-TMD-NBD	15	1108	121806.93	5.79	Plasma membrane
HvABCC12	A0A287KF05	TMD-NBD-TMD-NBD	30	1469	161017.89	7.21	Plasma membrane
HvABCC13	A0A287X7W7	TMD-NBD-TMD-NBD	12	1475	162032.55	8.14	Plasma membrane
HvABCC14	A0A287HCE6	TMD-NBD-TMD-NBD	11	1533	169070.73	6.33	Plasma membrane
HvABCC15	A0A287VRC4	TMD-NBD-TMD-NBD	13	1471	162585.72	6.93	Plasma membrane
HvABCC16	A0A287VRG5	TMD-NBD-TMD-NBD	13	1490	——	——	Plasma membrane
HvABCC17	A0A287M5V2	TMD-NBD-TMD-NBD	10	1496	167303.18	6.47	Plasma membrane
HvABCC	A0A287JB6	TMD-NBD-TMD-NBD	11	1538	171907	8.5	Plasma membrane

18	0				.93	1	
HvABCC 19	A0A287PXI 0	TMD-NBD-TMD-NBD	11	1442	159920 .68	8.1 6	Plasma membrane
HvABCC 20	A0A287KG2 5	TMD-NBD-TMD-NBD	10	1424	156262 .67	5.7 6	Plasma membrane
HvABCC 21	M0XGX5	TMD-NBD-TMD-NBD	10	1469	160306 .12	6.9 8	Plasma membrane
HvABCC 22	A0A287V R94	TMD-NBD-TMD-NBD	13	1485	164457 .69	8.0 5	Plasma membrane
HvABCD 1	F2D899	TMD-NBD	30	755	83879. 91	8.5 4	Extracellular
HvABCD 2	A0A287MQ F8	TMD-NBD	11	769	82097. 6	8.5 9	Plasma membrane
HvABCD 3	M0XWG4	TMD-NBD-TMD-NBD	27	1322	147939 .15	9.0 6	Extracellular
HvABCD 4	A0A287EH D3	TMD-NBD-TMD-NBD	25	1364	151499 .63	9.3 8	Extracellular
HvABCE 1	A0A287NT U8	NBD-NBD	12	590	66609. 64	6.3 2	Plasma membrane
HvABCE 2	A0A287JRE 8	NBD-NBD	11	611	68787. 52	7.6 8	Plasma membrane
HvABCE 3	A0A287FPU 6	NBD-NBD	12	636	71204. 23	8.9 5	Plasma membrane
HvABCF 1	M0V694	NBD-NBD	5	592	66031. 68	6.0 9	Cytoplasmic
HvABCF 2	M0WET2	NBD-NBD	5	595	66587. 25	6.3 7	Cytoplasmic
HvABCF 3	A0A287V1L 8	NBD-NBD	17	645	72014. 27	6.9 7	Cytoplasmic

HvABCF4	A0A287GMN2	NBD-NBD	2	733	——	——	Cytoplasmic
HvABCF5	A0A287DXM9	NBD-NBD	9	739	82742.73	8.86	Extracellular
HvABCG1	A0A287X8G4	NBD	1	171	19391.19	5.17	Plasma membrane
HvABCG2	A0A287K0G0	NBD	7	382	41160.22	5.16	Plasma membrane
HvABCG3	A0A287P766	NBD	1	390	42322.02	10.72	Plasma membrane
HvABCG4	A0A287QLG3	NBD	2	429	——	——	Vacuolar
HvABCG5	A0A287LF62	NBD	4	797	87161.29	10.16	Extracellular
HvABCG6	A0A287Y2H0	NBD	10	877	97605.42	9.12	Plasma membrane
HvABCG7	A0A287F5H3	NBD	15	1018	112603.18	8.86	Plasma membrane
HvABCG8	A0A287V6N4	NBD	14	1044	115199.45	8.69	Plasma membrane
HvABCG9	A0A287IQ01	NBD-TMD2	5	400	——	——	Plasma membrane
HvABCG10	A0A287H8V2	NBD-TMD2	7	511	——	——	Plasma membrane
HvABCG11	A0A287JT05	NBD-TMD2	2	554	60179.21	9.72	Plasma membrane
HvABCG12	A0A287L3B8	NBD-TMD2	1	608	65661.44	9.58	Vacuolar
HvABCG	A0A287NF	NBD-TMD2	4	642	68092.	8.9	Plasma membrane

13	M2				18	9	
HvABCG 14	F2DL63	NBD-TMD2	1	655	70966.09	8.71	Vacuolar
HvABCG 15	F2CQ28	NBD-TMD2	1	660	70844.8	8.94	Plasma membrane
HvABCG 16	F2DP21	NBD-TMD2	1	668	71944.43	9.35	Vacuolar
HvABCG 17	M0XY31	NBD-TMD2	1	681	73900.85	8.68	Vacuolar
HvABCG 18	M0YG47	NBD-TMD2	3	685	73122.92	9.59	Plasma membrane
HvABCG 19	F2DJI5	NBD-TMD2	8	689	76372.82	9.24	Plasma membrane
HvABCG 20	A0A287TNY7	NBD-TMD2	9	693	76032.84	9.34	Plasma membrane
HvABCG 21	M0YHK9	NBD-TMD2	11	723	79359.06	9.17	Plasma membrane
HvABCG 22	A0A287RFJ1	NBD-TMD2	4	725	81554.78	8.56	Vacuolar
HvABCG 23	A0A287XA03	NBD-TMD2	3	732	80841.39	9.06	Plasma membrane
HvABCG 24	A0A287RPK3	NBD-TMD2	8	755	82071.41	8.31	Plasma membrane
HvABCG 25	A0A287IWX2	NBD-TMD2	10	756	——	——	Plasma membrane
HvABCG 26	F2DQI5	NBD-TMD2	1	757	81278.75	9.14	Vacuolar
HvABCG 27	M0XE93	NBD-TMD2	9	757	82968.77	9.1	Plasma membrane

HvABCG28	A0A287ELL2	NBD-TMD2	1	789	85060.58	9.16	Vacuolar
HvABCG29	A0A287P7I6	NBD-TMD2	2	815	89076.67	9.1	Vacuolar
HvABCG30	A0A287W2R8	TMD2-PDR-NBD-TMD2	14	1111	125898.01	6.25	Plasma membrane
HvABCG31	A0A287THL9	TMD2-PDR-NBD	16	915	102841.07	7.61	Plasma membrane
HvABCG32	A0A287XC22	TMD2-PDR-NBD	15	943	104852.22	6.33	Plasma membrane
HvABCG33	A0A287T272	PDR-NBD-TMD2	24	759	85289.08	5.67	Plasma membrane
HvABCG34	A0A287R5Q3	NBD-TMD2-PDR-NBD-TMD2	23	1394	155669.69	9.16	Plasma membrane
HvABCG35	A0A287MTZ6	NBD-TMD2-PDR-NBD-TMD2	20	1265	144152.96	8.12	Plasma membrane
HvABCG36	A0A287VKI3	N-NBD-TMD2-PDR-NBD-TMD2	24	1392	156052.12	7.58	Plasma membrane
HvABCG37	A0A287WL54	N-NBD-TMD2-PDR-NBD-TMD2	23	1349	152825.44	8.8	Plasma membrane
HvABCG38	M0YEW0	N-NBD-TMD2-PDR-NBD-TMD2	22	1348	153853.13	8.09	Plasma membrane
HvABCG39	M0W6C2	N-NBD-TMD2-PDR-NBD-TMD2	24	1450	163114.82	6.86	Plasma membrane
HvABCG40	A0A287MJ91	N-NBD-TMD2-PDR-NBD-TMD2	23	1446	162239.64	7	Plasma membrane
HvABCG41	M0XGX1	N-NBD-TMD2-PDR-NBD-TMD2	23	1448	162481.79	6.19	Plasma membrane
HvABCG	A0A2R9J98	N-NBD-TMD2-PDR-NB	21	1447	161735	8.5	Plasma membrane

42	3	D-TMD2			.74	9	
HvABCG43	A0A287L2T7	N-NBD-TMD2-PDR-NBD-TMD2	21	1457	163941.76	7.36	Plasma membrane
HvABCG44	A0A287LKY2	N-NBD-TMD2-PDR-NBD-TMD2	23	1440	163209.36	7.32	Plasma membrane
HvABCG45	M0WIH1	N-NBD-TMD2-PDR-NBD-TMD2	19	1475	166619.47	6.85	Plasma membrane
HvABCG46	A0A287LHY7	N-NBD-TMD2-PDR-NBD-TMD2	22	1462	164749.92	7.48	Plasma membrane
HvABCG47	A0A287X8A3	N-NBD-TMD2-PDR-NBD-TMD2	21	1494	169476.99	8.33	Plasma membrane
HvABCG48	A0A287KEZ0	N-NBD-TMD2-PDR-NBD-TMD2	24	1431	161725.83	8.21	Plasma membrane
HvABCG49	A0A287SMH0	N-NBD-TMD2-PDR-NBD-TMD2	19	1496	168191.35	7.14	Plasma membrane
HvABCI1	A0A287VR45	NBD	2	175	19509.47	4.92	Plasma membrane
HvABCI2	F2DC59	NBD	1	224	25130.55	9.87	Plasma membrane
HvABCI3	M0X8C2	NBD	10	272	30599.4	9.18	Chloroplast
HvABCI4	A0A287LPQ4	NBD	5	289	31044.71	8.94	Chloroplast
HvABCI5	A0A287XIJ5	NBD	3	292	30868.57	6.35	Plasma membrane
HvABCI6	M0XST9	NBD	7	303	32484.17	6	Chloroplast
HvABCI7	A0A287PTL5	NBD	1	312	32846.68	6.18	Plasma membrane

HvABCI8	M0YCT8	NBD	11	387	41590.51	8.7	Chloroplast
HvABCI9	A0A287P1X9	NBD	7	402	44603.32	9.53	Cytoplasmic
